# Strength and conditioning practices of franchise-level cricket trainers

**DOI:** 10.17159/2078-516X/2020/v32i1a7786

**Published:** 2020-01-01

**Authors:** L Pote, G King, CJ Christie

**Affiliations:** 1 Department of Human Kinetics and Ergonomics, Rhodes University, Grahamstown, South Africa; 2 Cricket South Africa, Northlands, South Africa

**Keywords:** training, injury prevention, workload, monitoring

## Abstract

**Background:**

The purpose of this investigation was to determine the strength and conditioning practices implemented by cricket trainers and coaches at an elite level.

**Methods:**

An online survey, adapted from previous strength and conditioning questionnaires, was sent to trainers currently working with the franchise cricket teams in South Africa. The survey consisted of four main sections including a general strength and conditioning, cricket- specific and injury prevention category.

**Results:**

The results indicated that trainers (n = 5) implement planned sessions throughout the different phases of the season and that certain injury prevention practices are applied. Furthermore, player workload is monitored for all disciplines (batsmen, bowlers and fielders).

**Conclusion:**

These results can be used as a tool to educate coaches and trainers to ensure the correct strength and conditioning practices are implemented. Additionally the study showed that strength and conditioning practices at the elite level can be implemented without specialised equipment and facilities, which is important for teams that are constantly travelling. Lastly it showed the importance of looking at all aspects of health and skill related fitness.

The game of cricket is one of the oldest organised sports and is played throughout the world.^[1]^ It is constantly growing in popularity, particularly with the introduction and development of one-day and twenty/20 cricket.^[2]^ With these developments, the physical demands placed on the players are often substantial especially as individuals are often required to participate in multiple competitions, increasing the length of their seasons.^[3]^ Despite this, the research on how this has impacted the demands placed on players is still limited.^[3]^ It is important to understand these demands so that the appropriate strength and conditioning training and injury prevention practices can be implemented. Lately, there has been more interest in the physical demands of cricket with research focusing mainly on injuries.^[4]^ However, there is limited literature showing how strength and conditioning practices are being directed towards preparing cricketers for the demands imposed by the game. Furthermore, while our understanding of these demands has increased, if and how strength and conditioning trainers use this information is not known. Therefore, it is not clear how strength and conditioning trainers prepare players for the demands of the game.

Cricket places the body at risk as injuries in the sport are common.^[4]^ Fast bowlers are the most prone to injury, followed by batsmen and lastly fielders/wicketkeepers. Common injuries include stress fractures of the lumbar spine (fast bowlers), muscle strains of the lower limbs (batsmen) and impact injuries to the upper limbs (fielders/wicketkeepers).^[3]^ However, how strength and conditioning trainers use this information is not clear as there is limited literature on the strength and conditioning practices implemented by trainers to manage demands and reduce the risk of injury.^[3]^

Most of the research that has been done in the strength and conditioning field has looked at sports which have different demands to cricket.^[5]^ Only one study has examined the training practices of cricket trainers and this was limited to university and high school level trainers.^[1]^ There is limited literature on higher end trainers particularly at the professional level. Therefore, the purpose of this study was to determine the strength and conditioning practices that franchise level trainers implement at a professional level.

## Methods

### Experimental approach to the problem

This investigation was an online, survey-based, mixed-methods examination of the strength and conditioning practices of franchise level cricket trainers in South Africa. The survey required trainers to describe the practices they implement.

### Participants

The sample consisted of five head strength and conditioning trainers (age: 33 ± 3 years) from the different franchise-based cricket teams in South Africa. The small sample size and the fact that only male cricket trainers were investigated may be seen as a limitation of this study. Ethical clearance was granted by the Department of Human Kinetics and Ergonomics Ethical Standards Committee for research involving human participants (Rhodes University, Grahamstown, South Africa, RU-HSD-16-11-001). This included permission from Cricket South Africa (CSA). All trainers were informed of the benefits and risks of the investigation.

### Survey

The questionnaire was based on a previously validated cricket-specific survey that looked at the strength and conditioning practices of high school level trainers^[1]^ and consisted of three main sections:

1. General questions focusing on background information including age, gender, experience and qualifications.
2. Strength and conditioning questions looking specifically at physical testing as well as strength and conditioning for flexibility, speed, agility, plyometrics and strength development.
3. Cricket-specific questions examining the strength and conditioning practices for each specific discipline (bowling, batting and fielding/wicket-keeping).

Trainers were required to complete each question and could not proceed to the next question until the previous one had been completed. At the end of the survey a General Comments section was added where trainers were encouraged to mention any areas of interest that should be taken into account or considered from a strength and conditioning perspective.

### Procedures

An electronic letter describing the investigation was sent via email to the strength and conditioning trainers for the different franchise teams. This included an explanation of what the survey would entail and an electronic link (Google Forms) that the trainers needed to access in order to complete the survey. It was highlighted that by accessing the survey link, consent would be automatically given; however, all information would remain anonymous. Trainers were informed that they could exit the survey at any time without any further consequences. Lastly, it was specified that feedback would be provided via email if requested, but no trainer or franchise names would be included in the feedback response.

### Statistical analysis

The survey was made up of predominately fixed response (quantitative) questions. All responses were collated in real-time using an online survey platform (Google Forms) and exported to Microsoft Excel for further group and individual analyses. The descriptive statistics tool in Microsoft Excel was used to analyse the quantitative data. Only the “comments” section contained open-ended (qualitative) data. Here the data were collated and scrutinised for specific major and minor themes. This was completed by analysing data according to methods used in previous strength and conditioning surveys.^[5]^ These themes were then used to determine the results of this section.

## Results

### Background information

Absolute values were used to present the results. The basic demographic data of the trainers is displayed in Table 1.

### Physical testing

**Fig. 1:**
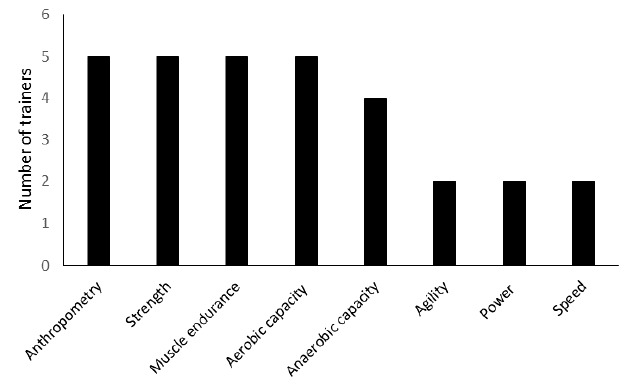
Physical testing parameters implemented by the trainers

All trainers indicated that their players were required to participate in some form of physical testing (Fig. 1). Every trainer completed the physical testing of their athletes in the preseason. In the in-season, four trainers indicated re-testing while in the off-season, three trainers indicated this. In the Yo-Yo Intermittent test, maximum push-ups in one minute and skinfold measures, were the most common tests used, followed by the 10-, 20- and 40 meter sprint test.

### Training practices and equipment

All of the trainers reported prescribing flexibility, agility, plyometrics and strength training during some of the season. Four out of the five did speed training. In terms of stretching, all of the trainers required players to stretch before (dynamic) and two after (static) a practice or match. Only one trainer required stretching at the gymnasium. The equipment and drills used by each trainer for the different training practices as well as the phase of season and sessions per week are described in Table 2.

### Cricket-specific conditioning and workload monitoring

Four out of the five trainers indicated that they implement different conditioning practices for each discipline (bowling, batting and fielding/wicket-keeping). For bowlers there was more focus on the posterior chain, the core, mobility and the lower body, whereas for batsmen there was a larger focus on the upper body, mobility and the core. There was no mention of specific conditioning practices for fielders.

In terms of monitoring, all of the trainers indicated that they implement some form of workload monitoring. This was through Cricket South Africa’s online monitoring platform, “Cricket Clinic”. The most common monitoring practices included the number of deliveries bowled, the load lifted, running intensity and distance, number of throws, average heart rate, as well as training load through session ratings of perceived exertion (sRPE) and duration.

### Injury monitoring and prevention

All of the trainers indicated that they kept a record of the injured players and the injuries they received. Three trainers specified that fast bowlers were the most prone to injury while two indicated that fielders were more susceptible to injury (these were fielders included in all the disciplines of the game). The most commonly injured areas for bowlers were the hamstrings and lower back region, whereas the shoulder was the most commonly injured area for fielders. Batsmen were most prone to impact injuries.

All the trainers indicated that they implemented some form of injury prevention exercises, mainly during the pre-season and in-season periods. Exercises included pre-habilitation exercises which focused on the main injuries that occurred (gluteus and deltoid muscles strengthening and stabilising), core work (prone bridge), hip flexor mobility and posterior chain strengthening. Lastly, in the comments section, it was noted that trainers prioritise strength over cardiovascular training and that players’ hip flexors and hamstrings are often tight due to prolonged periods of sitting (such as during travelling).

## Discussion

This is the first study that has examined the strength and conditioning practices implemented by trainers at a professional level. The most important finding was that there was planned strength and conditioning sessions in the different phases of competition. Further, that these trainers monitor workload and put in place injury prevention practices (pre-habilitation, core, mobility and posterior chain work) based on the injuries that occur for each discipline, something considered to be good practice.^[6]^ This is in contrast to a study done on trainers working with adolescent players at a high school and university level (non-professional players) where it was reported that this did not occur.^[1]^ Furthermore, all the trainers in the current investigation possessed some form of degree or diploma/certificate in strength and conditioning which was not the case at the adolescent level.^[1]^ Therefore, it would seem that trainer and coach education is important if the correct strength and conditioning and injury prevention practices are to be implemented.

When looking specifically at bowlers and batsmen, strengthening the posterior chain, core, mobility work (for bowlers), and upper body strength, mobility and core work (for batsmen) were the main focus. This is an important finding as previous research has indicated that upper body strength training, as well as core strengthening, is important for both injury prevention (particularly for the lower back) and improved performance.^[3,7,8]^ There were no reports of lower body injury prevention exercises. This is interesting considering that hamstring injuries (together with the lower back) were the most common injuries for fast bowlers (Table 4). There is extensive research showing that the strengthening and eccentric loading of the lower limb musculature (specifically the hamstrings) can reduce the risk of injury.^[9]^ While the trainers did implement box jumps, which are both concentrically and eccentrically focused, other injury prevention exercises that solely load the lower limbs eccentrically (such as Nordic hamstring lowers) may prevent injury through increased eccentric torque as well as the shifting of the torque joint angle curves of the hamstrings to longer muscle lengths.^[9]^ Lastly, while there was no mention of cricket-specific conditioning for fielders, the trainers did indicate that pre-habilitation exercises were implemented for shoulder injuries, which were the most prevalent for fielders and wicket-keepers respectively, a statement that previous literature supports.^[10,11]^

All the trainers indicated that they implemented some form of flexibility, plyometric, speed and agility training practices, mainly during the pre-season period (Table 2). These drills are important for injury prevention and improved performance.^[11–14]^ Furthermore, studies have shown that speed/acceleration and agility development for cricket players is important for aspects such as running between the wickets, as well as ground fielding and catching.^[14]^ However, if the components of health- and skill-related fitness are examined it would seem as if the trainers are not covering all aspects of preparation, particularly with regards to skills such as balance, coordination and reactions which are all important aspects required by cricket players. This is also evident in the parameters that are being tested at this level. It would appear that the trainers are testing the physical characteristics based on the most common, well known and validated tests rather than those specific to the game. Therefore, a specific test battery should be developed to guide programme design specific to the physical demands required in the game.

In terms of the equipment used for the development of particular physical characteristics (Table 2), most is non-specialised, cheap and cost-effective. This is specifically important for those non-professional teams that are restricted due to limited budgets and do not have access to specialised equipment or facilities, as well as when players are travelling or remotely located. Thus these types of characteristics can be developed anywhere without access to facilities with particular equipment that is inexpensive. However, the fact that specific exercises using the different types of equipment were not reported on is a limitation of this study, as this may have allowed a better understanding of the particular physical parameters being developed.

All the trainers did some form of workload monitoring for each of the different disciplines which has been linked to reduced injury risk in bowlers.^[15]^ It is also important to assist in identifying spikes in acute workload which can lead to overuse injuries that are becoming more prevalent in the modern day game.^[6,15]^ How these trainers use the workload information was not explicit and may be considered a limitation of this study. Effective use of the information is more important than the information itself.

### Summary and practical applications

Based on the findings of the current investigation it is important to:

- Ensure trainers possess appropriate strength and conditioning qualifications.
- Conduct a specific test battery, particularly in the pre-season period.
- Stretch before (dynamic) and after (static) a training session and match or perform mobility training to help with range of motion and flexibility.
- Develop physical attributes specifically flexibility, speed, agility and strength.
- Implement different training practices for each discipline (batsmen, bowlers and fielders/wicket keepers).
- To monitor the workload of each discipline to ensure a reduced risk of injury.

## Conclusion

The results of this study show the strength and conditioning practices of cricket trainers at a professional level, within a South African context. These can be used as a tool to educate trainers so that scientifically based strength and conditioning and injury prevention programmes can be implemented, particularly at a non-professional level where most trainers are lacking in these qualifications. Furthermore, it was shown that strength and conditioning can be implemented with limited equipment and facilities and can be translated to other levels of cricket, particularly at the non-professionals level. Therefore, the access to specialised facilities and equipment is not a necessity. From a practical perspective, this study demonstrates the importance of looking at most components of health and skill related fitness which can be used by the trainers at all levels. However, most of the data presented in this investigation are subjective and further studies should be conducted on larger sample groups and in other cricket playing nations.

## Figures and Tables

**Table 1:** Basic demographic data of the trainers

	n
**Gender (n=5)**	
Male	5
Female	0
**Experience (n=5)**	
> 5 years	3
< 5years	2
**Education (n=5)**	
Degree*	4
Diploma/certificate*	1

* Degree/diploma/certificate refers to a biokinetics, sports science or certified strength and conditioning specialist qualification as indicated by trainers. n, number of trainers

**Table 2:** Description of equipment, drills, the period of the season and the sessions per week for the different training practices

Type of training	Equipment (n)	Period of season (n)	Drills (n)	Sessions/ week (n)
Flexibility	Foam roller (5) R-band (5), Swiss ball (2)	Pre-season (5) In-season (5) Off-season (5)	Dynamic (5) Static (5) PNF (5) Ballistic (5)	N/A
Speed	Cones (5) Hurdles (5) R-bands (5) Weights (5) Sleds (5)	Pre-season (5) In-season (3) Off-season (4)	Sprint (5) Plyometrics (5) Resistance (5) Assistance (2)	1 (2) 2 (3)
Agility	Ladders (5) Cones (4) Hurdles (4) R-bands (3) Slalom poles (2)	Pre-season (5) In-season (2) Off-season (4)	N/A	1 (1) 2 (3) 5 (1)
Plyometrics	Boxes (5) Hurdles (5) Cones (2) R-bands (1) Medicine balls (1)	Pre-season (5) In-season (4) Off-season (4)	N/A	1 (1) 2 (3) 5 (1)
Strength	Dumbells (5) Barbells (5) Kettlebells (5) Body weight (5) Medicine balls (5) R-bands (4) Machines (3)	Pre-season (5) In-season (5) Off-season (5)	N/A	3 (4) 4 (1)

R-bands, resistance bands; N/A, not applicable; PNF, Proprioceptive neuromuscular facilitation; n, number of coaches
